# A Multi-Epitope FimH-CsgA-OmpA-Based Vaccine Induces Broad Anti-Adhesion Activity and Protects Chickens Against Avian Pathogenic *Escherichia coli* O2 Challenge

**DOI:** 10.3390/life16101586

**Published:** 2026-09-22

**Authors:** Jiayu Cui, Mingqing Shao, Long Wang, Ge Li, Jiantong Fang, Yan Ding, Weiwei Gao, PhyuHnin Wai, Yue Feng, Yingjie Pan, You Zhou, Chunhong Zhu, Qiangde Duan

**Affiliations:** 1 College of Veterinary Medicine, Yangzhou University, Yangzhou 225009, China; 2 Jiangsu Co-Innovation Center for Prevention and Control of Important Animal Infectious Diseases and Zoonoses/Joint Laboratory for International Cooperation in Agriculture and Agricultural Product Safety, Ministry of Education, Yangzhou 225009, China; 3 Jiangsu Joint Laboratory for International Cooperation in Prevention and Control of Important Animal Infectious Diseases and Zoonoses, Yangzhou 225009, China; 4 Jiangsu Institute of Poultry Science, Yangzhou 225125, China

**Keywords:** avian pathogenic *Escherichia coli*, multi-epitope fusion antigen, vaccine, immunogenicity, protection

## Abstract

Avian pathogenic *Escherichia coli* (APEC) causes substantial economic losses in the poultry industry, and vaccines capable of providing protection across diverse APEC strains remain needed. In this study, a FimH-CsgA-OmpA multiepitope fusion antigen (MEFA) was constructed and evaluated for immunogenicity, in vitro anti-adhesion activity, and protective efficacy against APEC challenge. Conserved immunodominant B-cell epitopes (BCEs) from CsgA and OmpA were incorporated into surface-exposed regions of a FimH scaffold. Structural modeling predicted that the inserted epitopes remained surface accessible without major disruption of the overall FimH-like fold, and immunoblotting confirmed that the recombinant fusion protein retained immunoreactivity with antibodies against FimH, CsgA, and OmpA. In mice, immunization with the fusion protein, either alone or formulated with Freund’s adjuvant, elicited FimH-, CsgA-, and OmpA-specific IgG responses and enhanced TNF-α and IL-6 production after antigen restimulation of splenocytes. Immune sera significantly inhibited the adhesion of APEC serotypes O1, O2, O8, O78, and O141 to DF-1 cells. In chickens, immunization induced FimH-, CsgA-, and OmpA-specific IgY responses. Following challenge with APEC-XM (O2), vaccinated chickens showed improved survival, reduced gross lesions, and a lower frequency of challenge-strain recovery from systemic tissues than PBS-treated controls. These findings demonstrate broad in vitro anti-adhesion activity across five APEC serotypes and protection against the tested APEC O2 challenge. Heterologous in vivo protection remains to be established.

## 1. Introduction

Avian pathogenic *Escherichia coli* (APEC), a major subgroup of extraintestinal pathogenic *Escherichia coli* (ExPEC), is one of the most important bacterial pathogens affecting the global poultry industry [1]. APEC infection causes avian colibacillosis, a systemic disease characterized by septicemia, airsacculitis, pericarditis, and perihepatitis, resulting in increased mortality, reduced production performance, carcass condemnation, and substantial economic losses [2]. Unlike intestinal pathogenic *E. coli*, APEC exhibits a complex pathogenic profile mediated by multiple virulence-associated determinants, including adhesins, iron acquisition systems, serum resistance factors, toxins, and invasion-related proteins [3,4,5]. These factors function cooperatively to facilitate bacterial colonization, immune evasion, and systemic dissemination. Moreover, APEC shares multiple virulence-associated determinants and pathogenic features with human ExPEC strains, highlighting its significance from both animal health and One Health perspectives [6,7]. The extensive genetic and phenotypic diversity of APEC populations remains a major obstacle to the development of effective vaccines capable of providing broad protection.

Antimicrobial therapy remains an important strategy for controlling APEC-associated diseases; however, the intensive use of antibiotics in poultry production has accelerated the emergence of multidrug-resistant strains and increased concerns regarding antimicrobial resistance transmission [8,9]. Vaccination represents a sustainable approach to reduce antibiotic dependency and prevent avian colibacillosis. Various vaccine platforms, including whole-cell vaccines, attenuated vaccines, and recombinant subunit vaccines, have been investigated [10,11,12]. Nevertheless, the protective efficacy of current vaccines is frequently limited by the substantial antigenic diversity and genetic variability of APEC strains. Although serotypes O1, O2, and O78 are commonly associated with clinical outbreaks, vaccines targeting individual serotypes or single antigens often provide insufficient cross-protection against diverse field isolates [13]. Therefore, developing a safe and broadly protective vaccine remains a critical objective in APEC vaccinology.

Subunit vaccines targeting conserved virulence-associated proteins have emerged as attractive candidates due to their safety, defined composition, and potential for rational antigen optimization. Among various virulence determinants, adhesion-associated proteins are particularly promising vaccine targets because bacterial attachment represents a critical initial step in APEC colonization and infection [3,14]. FimH, the mannose-specific adhesin located at the distal tip of type I fimbriae, mediates the initial interaction between bacteria and host cells and contributes to APEC colonization [15]. CsgA, the major structural component of curli fimbriae, participates in bacterial adhesion, biofilm formation, and environmental persistence [16]. OmpA, a highly conserved outer membrane protein, contributes to bacterial adhesion, host interaction, and adaptation during infection [17,18]. Importantly, these adhesion-associated proteins are widely conserved among pathogenic *E. coli* strains, supporting their potential application as vaccine antigens. However, most existing APEC subunit vaccines have focused on individual antigens, which may not adequately address the multifactorial nature of APEC pathogenesis [10,19,20]. Because successful infection involves the coordinated activity of multiple virulence factors, single-antigen vaccines may induce incomplete immune responses and provide limited protection against genetically diverse strains. Thus, whether the combination of conserved protective epitopes from multiple virulence-associated proteins can enhance immune breadth and protective efficacy remains an important unresolved question.

The multi-epitope fusion antigen (MEFA) platform provides a rational strategy to overcome these limitations by enabling the simultaneous presentation of multiple immunodominant epitopes within a single recombinant antigen [21,22]. Previous studies have demonstrated the potential of MEFA-based approaches for developing subunit vaccines against *E. coli* and other Gram-negative bacterial pathogens through the incorporation of multiple protective epitopes into a unified antigenic construct [23,24,25,26]. Particularly, MEFA technology provides a framework for combining conserved protective determinants from distinct virulence-associated proteins to overcome antigenic variability. Compared with conventional single-antigen vaccines, MEFA-based vaccines allow the integration of functionally complementary epitopes while maintaining antigen stability and facilitating recombinant production [22]. Through rational epitope selection and optimized antigen presentation, MEFA technology may enhance immune recognition and promote balanced humoral and cellular immune responses against complex bacterial pathogens.

The objective of this study was to construct a FimH-CsgA-OmpA multiepitope fusion antigen incorporating conserved B-cell epitopes from CsgA and OmpA into a FimH scaffold and to evaluate its immunogenicity, antibody-mediated anti-adhesion activity, and protective efficacy against APEC in mouse and chicken models.

## 2. Materials and Methods

### 2.1. Bacterial Strains, Plasmids, and Cell Line Culture Conditions

The APEC-XM strain (O2) was kindly provided by Professor Chengping Lu (Nanjing Agricultural University). The APEC2411 (O1), APEC2431 (O8), APEC2448 (O78), and APEC2472 (O141) strains were isolated and maintained in the laboratory. The APEC-XM strain and the four clinical isolates were used in in vitro bacterial adherence inhibition assays. The expression vector pET28α+ (Novagen, Madison, WI, USA) and *E. coli* strains Top10 and BL21 (DE3) were used for gene cloning and recombinant protein expression. The chicken embryo fibroblast cell line DF-1 was cultured in Dulbecco’s modified Eagle medium (DMEM) supplemented with 10% heat-inactivated fetal bovine serum (FBS; Gibco, Thermo Fisher Scientific, Grand Island, NY, USA) and maintained in a humidified atmosphere containing 5% CO_2_ at 37 °C.

### 2.2. Construction and Bioinformatics Analysis of the FimH-CsgA-OmpA MEFA Fusion Protein

Candidate dominant and conserved linear B-cell epitopes (BCEs) derived from CsgA and OmpA were identified through a multi-step bioinformatics pipeline. Initially, linear BCEs were predicted using BepiPred 2.0 (Technical University of Denmark, Kongens Lyngby, Denmark, and La Jolla Institute for Allergy and Immunology, La Jolla, CA, USA; 2017), and residues with epitope probability scores >0.5 were considered potential epitope candidates. Contiguous regions comprising at least seven consecutive residues exceeding this threshold were selected as putative BCEs. Subsequently, conservation analysis was performed using representative CsgA and OmpA sequences from different O serotypes of APEC strains. The conservation levels of candidate epitopes were quantitatively assessed, and highly conserved regions were selected for subsequent construct design. The selected epitopes were then rationally incorporated into surface-exposed loops of the FimH scaffold to generate the FimH-CsgA-OmpA MEFA fusion protein. The antigenicity, physicochemical properties, and allergenicity of the resulting fusion protein were further evaluated using VaxiJen v2.0 (Drug Design and Bioinformatics Lab, Faculty of Pharmacy, Medical University of Sofia, Sofia, Bulgaria; server originally described in 2007), ExPASy ProtParam (SIB Swiss Institute of Bioinformatics, Lausanne, Switzerland; resource described by Gasteiger et al., 2005), and AllerTOP v2 (Faculty of Pharmacy, Medical University of Sofia, Sofia, Bulgaria; 2014), respectively. Finally, structural modeling with AlphaFold 3 (Google DeepMind and Isomorphic Labs, London, UK; 2024) was used to assess the predicted spatial accessibility and surface exposure of the inserted BCEs within the FimH scaffold.

### 2.3. Construction of the Recombinant Plasmids

The pUC57-fimH-csgA-ompA plasmid was synthesized and used as a template for PCR amplification of the *fimH-csgA-ompA* gene fragment using the primers *fimH*-F and *fimH*-R (Table 1). Genomic DNA extracted from the APEC-XM strain was used as a template for PCR amplification of the *fimH*, *csgA*, and *ompA* genes. The specific primers used for amplification of these genes are listed in Table 1. The amplified *fimH-csgA-ompA*, *fimH*, and *csgA* gene fragments were cloned into the *Bam*HI/*Sal*I-digested pET28α+ vector, whereas the amplified *ompA* gene fragment was cloned into the *Nhe*I/*Sal*I-digested pET28α+ vector to generate the corresponding recombinant plasmids.

### 2.4. Expression and Identification of Recombinant Proteins

The FimH-CsgA-OmpA MEFA fusion protein and the FimH, CsgA, and OmpA recombinant proteins were expressed and purified as previously described [27]. Briefly, a single recombinant *E. coli* colony containing each construct was grown overnight at 37 °C with vigorous shaking at 220 rpm in 2× yeast extract tryptone (2×YT) medium supplemented with 30 μg/mL kanamycin. The following day, the overnight culture was subcultured into 500 mL of fresh 2×YT medium containing 30 μg/mL kanamycin at a 1:100 dilution ratio. When the optical density at 600 nm (OD600) reached approximately 0.6, recombinant protein expression was induced by adding 1 mM isopropyl-β-D-1-thiogalactoside (IPTG), followed by incubation for an additional 4 h. The bacterial cells were then harvested by centrifugation at 12,000 rpm for 15 min. Total recombinant proteins were extracted by sonication and subsequently purified from the total protein extracts using Protino^®^ Ni-TED 2000 packed columns (MACHEREY-NAGEL, Germany) according to the manufacturer’s instructions. Purified recombinant proteins were analyzed by 12% sodium dodecyl sulfate–polyacrylamide gel electrophoresis (SDS-PAGE) followed by Coomassie Brilliant Blue staining. The FimH-CsgA-OmpA fusion protein was further analyzed by western blotting using mouse anti-FimH serum (1:6000), anti-CsgA serum (1:7000), and anti-OmpA serum (1:10,000). Additionally, the FimH, CsgA, and OmpA recombinant proteins were identified using mouse anti-FimH-CsgA-OmpA serum (1:7000).

### 2.5. Mouse Immunization

Animal experiments were approved by and conducted at the Animal Experiment Center of Yangzhou University (Yangzhou, China) in accordance with protocols approved by the Institutional Animal Care and Use Committee of Yangzhou University (Approval No. YZU-IACUC-202402079). Twenty-four 6-week-old specific pathogen-free (SPF) BALB/c mice were randomly assigned to three groups (*n* = 8 per group): the PBS control group, the FimH-CsgA-OmpA group, and the FimH-CsgA-OmpA+Freund’s adjuvant (FA) group. After an acclimatization period of 1–2 days, mice in each group were immunized by multiple subcutaneous injections at the dorsal neck region. Mice in the FimH-CsgA-OmpA group received the fusion protein without adjuvant, whereas those in the PBS control group received an equal volume of sterile PBS. For the FimH-CsgA-OmpA+FA group, the recombinant protein was emulsified with an equal volume of complete Freund’s adjuvant (CFA) prior to immunization. The immunization dose was 50 μg of protein per mouse for all immunized groups. Booster immunizations were administered at two-week intervals, for a total of three immunizations. The same immunization dose was used for each booster, and incomplete Freund’s adjuvant (IFA) was used instead of CFA for booster immunizations in the adjuvant group. Blood samples were collected weekly before primary immunization and after the first immunization. On day 42, all mice were euthanized, and splenocytes were harvested for subsequent cytokine analysis.

### 2.6. Chicken Immunization

Eighty 5-day-old SPF White Leghorn chicks were supplied by Sinopharm Group Yangzhou Weike Biological Engineering Co., Ltd. and randomly assigned to four groups (*n* = 20 per group): the FimH-CsgA-OmpA+MOA group, the FimH-CsgA-OmpA fusion antigen group, the PBS group, and the mock group. The chicks were acclimatized until 7 days of age before immunization. Chicks in the immunization groups received multiple subcutaneous injections at the dorsal neck region, with an immunization dose of 500 μg per chick. In the FimH-CsgA-OmpA+MOA group, the recombinant protein was emulsified with white oil adjuvant at a protein-to-adjuvant volume ratio of 2:1 (*V*/*V*) before administration. Chicks in the FimH-CsgA-OmpA group received 500 μg of the fusion protein without adjuvant, whereas those in the PBS control groups received an equal volume of sterile PBS at the same injection site. The mock group remained unvaccinated and unchallenged. Primary immunization was performed at 7 days of age, followed by booster immunization at 28 days of age using the same route and dosage. Chickens were individually weighed, and body weights were recorded at 7, 14, 21, 28, and 35 days of age. Blood samples were collected at the corresponding time points for serum separation.

### 2.7. ELISAs for the Detection of Anti-FimH, Anti-CsgA, and Anti-OmpA Antibody Titers

ELISAs were performed to quantify mouse serum IgG and chicken serum IgY titers specific to FimH, CsgA, and OmpA, as previously described [28]. Briefly, purified recombinant FimH, CsgA, or OmpA protein was used as the coating antigen, and 500 ng of each protein was added to each well of a 96-well plate. The uncoated sites were blocked with 10% non-fat milk at 37 °C for 1 h, followed by three washes with 0.05% PBST (1× PBS containing 0.05% Tween-20). Mouse or chicken serum samples were serially diluted two-fold from 1:200 to 1:12,800 and analyzed by ELISA. Horseradish peroxidase (HRP)-conjugated goat anti-mouse IgG (1:5000) or HRP-conjugated goat anti-chicken IgY (1:5000) was used as the secondary antibody. The OD450 value of each well was measured after incubation with 3,3′,5,5′-tetramethylbenzidine (TMB) HRP color development solution at 37 °C for 30 min. The reaction was terminated by adding 2 M H_2_SO_4_. All experiments were performed in triplicate to ensure the reliability and reproducibility of the results. Antibody titers were defined as the highest serum dilution that produced an OD450 value greater than 0.3 after background subtraction and were subsequently subjected to log10 transformation.

### 2.8. Cytokine Production Analysis in Mouse Splenocytes Following Antigen Stimulation

Two weeks after the final immunization, spleens were aseptically collected from immunized mice. Splenocytes were isolated by mechanical disruption and filtration through a cell strainer, followed by erythrocyte lysis. The resulting single-cell suspensions were resuspended in RPMI 1640 medium supplemented with 0.1% FBS and 1% penicillin–streptomycin, and the cell concentration was adjusted to 2.5 × 10^5^ cells/mL. Cells were seeded into six-well culture plates at a density of 5 × 10^5^ cells per well and stimulated with 100 µg of purified recombinant FimH-CsgA-OmpA protein. Following incubation in a humidified CO_2_ incubator for 72 h, culture supernatants were collected, centrifuged to remove cellular debris, and stored for subsequent cytokine analysis. The levels of tumor necrosis factor-α (TNF-α) and interleukin-6 (IL-6) in the supernatants were quantified using commercially available mouse ELISA kits according to the manufacturers’ instructions. All assays were performed in triplicate, and cytokine concentrations were calculated based on standard curves generated for each plate.

### 2.9. Antibody-Mediated Inhibition of APEC Adhesion to DF-1 Cells

An in vitro antibody-mediated bacterial adhesion inhibition assay was performed using DF-1 cells and wild-type APEC strains, including APEC2411 (O1), APEC-XM (O2), APEC2431 (O8), APEC2448 (O78), and APEC2472 (O141), with minor modifications from a previously described method [29]. Briefly, DF-1 cells were seeded into 96-well plates at a density of approximately 5 × 10^4^ cells per well and cultured overnight to establish a confluent monolayer. APEC strains were cultured overnight in LB broth at 37 °C with shaking and subsequently subcultured at a 1:100 ratio into fresh antibiotic-free LB broth for an additional 2 h at 37 °C with agitation (220 rpm). The bacterial concentration was determined based on OD600 measurements and adjusted to 1 × 10^7^ CFU/mL using sterile PBS.

For the adhesion inhibition assay, mouse serum (40 μL), bacterial suspension (200 μL), and DMEM (160 μL) were mixed and incubated at room temperature with gentle agitation (80 rpm) for 30 min. The DF-1 cell monolayers were washed three times with sterile PBS and then incubated with 100 μL of the serum-bacteria mixture at 37 °C for 1 h. After incubation, the supernatants were removed, and the cells were washed three times with sterile PBS to remove non-adherent bacteria. The cells were subsequently treated with 100 μL of trypsin and incubated at 37 °C for 10 min to detach the cells and recover cell-associated bacteria. The recovered cell suspensions were transferred into sterile tubes containing 900 μL of PBS and serially diluted. Aliquots (10 μL) of each dilution were plated onto LB agar plates in triplicate and incubated at 37 °C for 16 h. Bacterial colony-forming units (CFUs) were counted to determine the adhesion capacity of APEC strains.

### 2.10. Chicken Challenge Trial

A chicken challenge trial was conducted to evaluate the protective efficacy of the FimH-CsgA-OmpA MEFA vaccine against APEC infection. At 35 days of age, chickens were intratracheally inoculated with 0.2 mL of the virulent APEC O2 strain APEC-XM containing 1 × 10^9^ CFU per bird. Seven days post-challenge, surviving chickens were humanely euthanized, and necropsy examinations were performed to evaluate gross pathological lesions in the lungs, heart, liver, and spleen.

The severity of lesions was independently scored by at least two veterinarians according to the scoring system established by Antão et al. To determine APEC bacterial burdens in major organs, tissue samples from the lungs, heart, liver, and spleen were aseptically collected and maintained on ice. The collected tissues were weighed, homogenized in sterile PBS, serially diluted, and plated onto MacConkey agar. After overnight incubation at 37 °C, bacterial colonies were enumerated, and APEC loads were calculated and expressed as colony-forming units per gram of tissue (CFU/g). Representative colonies recovered from MacConkey agar plates were identified by PCR using O2 serotype-specific primers (Table 1) to confirm their identity as the challenge strain.

### 2.11. Statistical Analysis

All data are presented as means ± standard deviations (SD) and were analyzed using GraphPad Prism version 9.0. Comparisons between two groups were performed using Student’s *t*-test, while multiple-group comparisons were conducted using one-way analysis of variance (ANOVA). Statistical significance was defined as *p* < 0.05, with *p* < 0.01 considered highly significant.

## 3. Results

### 3.1. Design and Bioinformatics Analysis of the FimH-CsgA-OmpA MEFA Fusion Protein

Four predicted non-toxic and immunodominant B-cell epitopes (BCEs) derived from CsgA (amino acid positions 76–89 and 96–112) and OmpA (amino acid positions 126–141 and 246–276) were selected to replace the low-immunogenic antigenic regions exposed on the surface of the FimH scaffold protein, resulting in the construction of the FimH-CsgA-OmpA MEFA (Figure 1A). The predicted secondary structure of the fusion protein was primarily composed of ordered α-helices and β-strands. The inserted epitopes were seamlessly integrated into the FimH scaffold, maintaining structural continuity (Figure 1B). The predicted three-dimensional structure demonstrated that the fusion protein adopted a compact and stable conformation with a topology comparable to that of native FimH (Figure 1C). No apparent structural disruption, abnormal folding, or extensive disordered regions were observed. Notably, the inserted CsgA- and OmpA-derived epitopes were predominantly exposed on the protein surface, suggesting their potential accessibility for immune recognition.

The physicochemical properties and immunological characteristics of the FimH-CsgA-OmpA fusion protein were further evaluated using bioinformatics analyses. The fusion protein consisted of 328 amino acids, with a predicted molecular weight of 34.6 kDa. The instability index was 22.2 (<40), consistent with a stable protein prediction, and the grand average of hydropathicity (GRAVY) was −0.054, indicating slight overall hydrophilicity. Furthermore, the fusion protein was predicted to be non-toxic and non-allergenic. The predicted protective antigenicity score was 0.7919, exceeding the threshold value of 0.4, indicating strong antigenic potential. Collectively, these results demonstrate that the FimH-CsgA-OmpA MEFA fusion protein possesses favorable structural stability, safety characteristics, and antigenic properties, supporting its further evaluation as a potential vaccine candidate.

### 3.2. Expression and Identification of the FimH-CsgA-OmpA MEFA Fusion Protein

The FimH-CsgA-OmpA MEFA fusion protein, together with the recombinant FimH, CsgA, and OmpA proteins, was successfully expressed, extracted, and purified. SDS-PAGE analysis with Coomassie brilliant blue staining revealed that the FimH-CsgA-OmpA MEFA fusion protein and the recombinant FimH, CsgA, and OmpA proteins exhibited molecular masses of approximately 35, 31, 37, and 15 kDa, respectively, consistent with their predicted molecular weights (Figure 2A). Western blot analysis showed that the FimH-CsgA-OmpA MEFA fusion protein was specifically recognized by polyclonal antibodies against FimH, CsgA, and OmpA (Figure 2B–D). Conversely, antibodies raised against the FimH-CsgA-OmpA fusion protein specifically reacted with the recombinant FimH, CsgA, and OmpA proteins, respectively (Figure 2E–G). These results demonstrate that the dominant BCEs derived from CsgA and OmpA were successfully presented on the FimH scaffold protein while preserving the native antigenic characteristics of FimH.

### 3.3. Immunization of Mice with the FimH-CsgA-OmpA MEFA Fusion Protein Elicits Anti-FimH, Anti-CsgA, and Anti-OmpA IgG Antibodies

The levels of anti-FimH, anti-CsgA, and anti-OmpA IgG antibodies in mouse sera were measured, and the results are presented in Figure 3. Immunization with the FimH-CsgA-OmpA fusion protein, either alone or formulated with FA, elicited specific IgG responses against FimH, CsgA, and OmpA. Kinetic analysis revealed that antigen-specific antibody levels increased within one week after primary immunization and further increased following booster immunizations, reaching the highest levels at the final serum collection time point (Figure 3A,B). Analysis of terminal serum samples showed that antigen-specific IgG antibody levels were slightly higher in the FimH-CsgA-OmpA+FA group than in the FimH-CsgA-OmpA group; however, this difference did not reach statistical significance (Figure 3C). In contrast, no FimH-, CsgA-, or OmpA-specific antibodies were detected in the PBS control group. Collectively, these results demonstrate that the FimH-CsgA-OmpA fusion protein exhibits robust immunogenicity and induces simultaneous humoral immune responses against multiple target antigens, including FimH, CsgA, and OmpA.

### 3.4. Immunization with the FimH-CsgA-OmpA MEFA Fusion Protein Induces Antigen-Specific Cytokine Responses

Splenocytes isolated from immunized mice were cultured and restimulated in vitro with the recombinant FimH-CsgA-OmpA MEFA fusion protein, and the concentrations of TNF-α and IL-6 in culture supernatants were determined by ELISA. As shown in Figure 4, antigen stimulation significantly increased TNF-α (Figure 4A) and IL-6 (Figure 4B) production in splenocytes from mice immunized with either the FimH-CsgA-OmpA+FA or FimH-CsgA-OmpA formulation compared with the PBS control group (*p* < 0.01). In both immunized groups, in vitro restimulation with the FimH-CsgA-OmpA MEFA fusion protein resulted in significantly elevated levels of TNF-α and IL-6 compared with the corresponding unstimulated controls (*p* < 0.01). Furthermore, the FimH-CsgA-OmpA+FA group exhibited significantly higher TNF-α and IL-6 production than the FimH-CsgA-OmpA group (*p* < 0.01). Collectively, these findings demonstrate that immunization with the FimH-CsgA-OmpA MEFA fusion protein induces potent antigen-specific cytokine responses, which are further enhanced by formulation with FA.

### 3.5. Serum from Immunized Mice Inhibits APEC Adherence to DF-1 Cells

The inhibitory effects of sera from immunized mice on APEC adherence were evaluated in vitro using a DF-1 cell model. APEC strains of serotypes O1, O2, O8, O78, and O141 were pre-incubated with pooled sera from mice immunized with FimH-CsgA-OmpA+FA or FimH-CsgA-OmpA and subsequently assessed for their ability to adhere to DF-1 cells. All five APEC strains exhibited significantly reduced adherence following incubation with sera from either immunized group compared with the PBS control serum group (*p* < 0.01) (Figure 5). However, no significant difference in adherence was observed between the FimH-CsgA-OmpA+FA and FimH-CsgA-OmpA groups. These findings indicate that immunization with the FimH-CsgA-OmpA vaccine candidate induces antibodies that significantly inhibit the adherence of multiple APEC serotypes to DF-1 cells.

**Figure 5:**
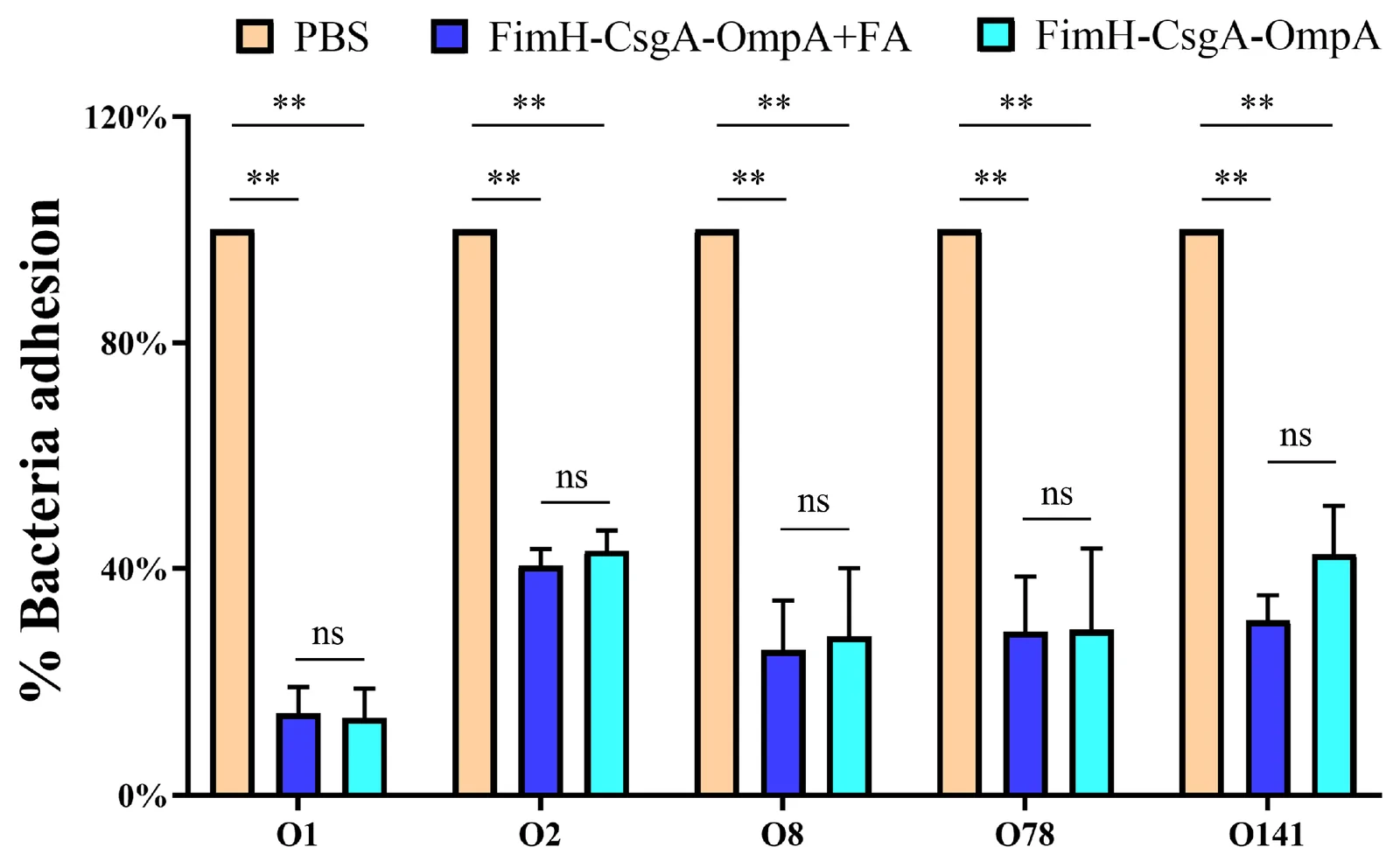
**Inhibition of APEC adherence to DF-1 cells by sera from immunized mice.** APEC strains of serotypes O1, O2, O8, O78, and O141 were pre-incubated with pooled sera from mice immunized with FimH-CsgA-OmpA+FA, FimH-CsgA-OmpA, or PBS and subsequently assessed for their ability to adhere to DF-1 cells. Sera from both immunized groups significantly reduced the adherence of all tested APEC strains compared with PBS control sera, whereas no significant difference was observed between the FimH-CsgA-OmpA+FA and FimH-CsgA-OmpA groups. Data are presented as mean ± SD. ns, not significant (*p* > 0.05). **: *p*< 0.01.

### 3.6. Immunization of Chickens with the FimH-CsgA-OmpA MEFA Fusion Protein Elicits FimH-, CsgA-, and OmpA-Specific IgY Antibodies

To evaluate the humoral immune response elicited by the FimH-CsgA-OmpA MEFA fusion protein in chickens, serum samples were collected at 7, 14, 21, and 28 days post-primary immunization, and antigen-specific IgY levels were monitored longitudinally. One week after primary immunization, FimH-, CsgA-, and OmpA-specific IgY antibodies were detectable in sera from chickens immunized with the adjuvanted fusion protein (Figure 6A) or the fusion protein alone (Figure 6B). Following booster immunization, serum IgY levels increased progressively in both immunized groups and remained elevated throughout the observation period. Analysis of terminal serum samples demonstrated that the levels of FimH-, CsgA-, and OmpA-specific IgY antibodies (Figure 6C) were significantly higher in both immunized groups than in the PBS control group. Furthermore, antibody levels were significantly higher in the adjuvanted group than in the non-adjuvanted group, indicating that adjuvant formulation enhanced the humoral immune response. Collectively, these findings indicate that the FimH-CsgA-OmpA MEFA fusion protein is highly immunogenic and elicits robust antibody responses against FimH, CsgA, and OmpA.

### 3.7. Immunization Improves Survival of Chickens Following APEC Challenge

As shown in Figure 7, no mortality was observed in the mock group throughout the observation period, with a final survival rate of 100%. In contrast, chickens in the PBS control group exhibited typical clinical signs after challenge, including depression, ruffled feathers, and reduced feed and water intake. Mortality was first observed at 24 h post-challenge, and the cumulative survival rate declined to 50% by the end of the observation period. Chickens immunized with the FimH-CsgA-OmpA fusion protein alone showed 100% survival throughout the 7-day observation period. In the FimH-CsgA-OmpA+MOA group, mortality was observed at 48 h post-challenge, resulting in a final survival rate of 83%. Kaplan–Meier survival analysis using the log-rank test demonstrated significantly improved survival in both immunized groups compared with the PBS control group. Thus, immunization with the FimH-CsgA-OmpA fusion protein improved survival following challenge with the tested APEC-XM (O2) strain.

### 3.8. Immunization Alleviates Organ Lesions and Limits APEC-XM Recovery in Challenged Chickens

As shown in Figure 8A, challenge with the APEC-XM (O2) strain resulted in sporadic lesions in the heart and liver, accompanied by marked opacity of the thoracic and abdominal serosal surfaces and deposition of pale yellow to milky-white fibrinous exudates. No apparent lesions were observed in the lungs or spleens. Vaccination substantially attenuated gross pathological lesions, and no apparent lesions were observed in the lungs or spleens of either vaccinated group. As expected, no gross pathological lesions were detected in the examined organs of the mock group. Because histopathological examination was not performed, these observations are limited to gross pathology.

Bacterial isolation was performed using blood and major organ samples (lungs, heart, liver, and spleen) collected from chickens that died during the observation period and those that survived until the end of the experiment. APEC-XM was recovered from the blood and major organs of chickens that died following challenge, whereas no APEC-XM was recovered from any samples collected from surviving chickens (Table 2). The proportion of chickens positive for APEC-XM recovery was 0% in the mock group, 50% in the PBS control group, 17% in the FimH-CsgA-OmpA+MOA group, and 0% in the FimH-CsgA-OmpA group (Figure 8B). PCR analysis of the recovered isolates revealed a specific amplicon of approximately 665 bp in all isolates, corresponding to the expected size of the APEC-XM-specific target fragment (Figure 8C). Collectively, these findings demonstrate that immunization with the FimH-CsgA-OmpA MEFA fusion protein conferred protection against APEC-XM challenge by reducing gross pathological lesions and preventing bacterial recovery from systemic tissues of surviving chickens.

## 4. Discussion

APEC remains a major bacterial pathogen threatening poultry production worldwide due to its diverse virulence repertoire, extensive genetic heterogeneity, and ability to cause systemic infections. Although vaccination represents a promising strategy for controlling avian colibacillosis and reducing antimicrobial usage, the development of broadly protective vaccines remains challenging because APEC pathogenicity involves multiple virulence determinants and circulating strains exhibit considerable antigenic diversity [1,30,31,32]. The present study evaluated a MEFA vaccine based on three conserved adhesion-associated proteins, FimH, CsgA, and OmpA. This vaccine induced broad antigen-specific immune responses, and its antibodies inhibited adhesion of five APEC serotypes in vitro. In the chicken model, protection was demonstrated against the tested APEC-XM O2 challenge. These findings support the feasibility of integrating multiple conserved protective epitopes into a single recombinant antigen while highlighting the need for heterologous challenge studies.

The successful construction and antigenic characterization of the FimH-CsgA-OmpA MEFA fusion protein represent an important first step toward rational APEC vaccine development. In this study, immunodominant BCEs derived from CsgA and OmpA were selected through bioinformatics prediction and conservation analysis and subsequently incorporated into surface-exposed regions of the FimH scaffold. Structural modeling demonstrated that the inserted epitopes were accessible on the protein surface without causing major conformational disruption of the FimH backbone. Furthermore, western blot analysis confirmed that the fusion protein retained antigenic recognition by antibodies against FimH, CsgA, and OmpA, indicating that the incorporated epitopes preserved their native antigenic properties. These findings are consistent with previous studies demonstrating that MEFA technology can effectively present multiple protective epitopes while maintaining antigen stability and immunogenicity [23,33,34]. Unlike traditional recombinant subunit vaccines that usually focus on a single virulence factor, the MEFA strategy used in this study enables simultaneous targeting of multiple bacterial components involved in infection, thereby potentially improving immune coverage against genetically diverse APEC populations.

Adhesion-associated proteins were selected as vaccine targets because bacterial attachment represents an essential early event in APEC colonization and systemic infection. FimH-mediated adhesion through type I fimbriae facilitates the initial interaction between bacteria and host epithelial cells, whereas CsgA contributes to curli formation, biofilm development, and bacterial persistence [15,16]. OmpA, as a highly conserved outer membrane protein, participates in host-pathogen interactions and contributes to bacterial adaptation during infection [17,18]. Previous studies have demonstrated that antibodies targeting individual adhesion-related proteins can interfere with bacterial attachment and provide partial protection against *E. coli* infection. However, because APEC pathogenicity relies on coordinated functions of multiple virulence factors, targeting a single antigen may not provide sufficient immune pressure against heterogeneous strains. The present study extends previous findings by combining protective epitopes from three adhesion-associated antigens into one fusion construct, thereby providing simultaneous immune recognition of multiple conserved virulence determinants.

The immunological results obtained in the mouse model further demonstrated the strong immunogenic potential of the FimH-CsgA-OmpA MEFA vaccine. Immunization induced robust IgG responses against FimH, CsgA, and OmpA, indicating that the engineered fusion protein successfully preserved the antigenicity and immunogenicity of the parental proteins. Notably, comparable antigen-specific antibody responses were observed between mice immunized with the FimH-CsgA-OmpA fusion protein alone and those receiving Freund’s adjuvant, which is consistent with, but does not establish, intrinsic immunostimulatory activity of the construct. FimH has been reported to exhibit adjuvant-like properties in other experimental contexts [35,36]. In particular, FimH promoted dendritic-cell maturation and antigen-specific T-cell activation through a TLR4-dependent but MD-2-independent mechanism in a cancer-immunotherapy model [35], and FimH derivatives have enhanced immune responses in other vaccine settings [36,37,38]. Because TLR4 can signal through MyD88- and TRIF-dependent pathways, downstream NF-κB- and IRF3-associated programs provide a plausible framework for FimH-associated innate immune activation. However, these pathways were not examined in the present vaccine system. Direct FimH–TLR4 interaction, dendritic-cell/APC maturation, MHC or costimulatory-molecule expression, and MyD88/TRIF signaling were not measured. Therefore, the similar antibody responses observed with and without Freund’s adjuvant should be interpreted only as being compatible with intrinsic immunostimulatory activity, not as evidence of a defined FimH–TLR4 mechanism. Dedicated mechanistic studies will be required to determine how FimH contributes to innate sensing and adaptive immune priming in this construct. In addition to strong humoral responses, vaccination stimulated antigen-specific cellular immune responses, as demonstrated by increased production of TNF-α and IL-6 following antigen restimulation. Although antibody-mediated inhibition of adhesion represents a major protective mechanism against extracellular bacterial pathogens, enhanced cellular immune activation may further contribute to bacterial clearance by promoting inflammatory responses and immune coordination.

A particularly important finding of this study was the broad inhibitory activity of vaccine-induced antibodies against multiple APEC serotypes. Serum antibodies from immunized mice significantly reduced the adherence of APEC strains belonging to serotypes O1, O2, O8, O78, and O141 to DF-1 cells. The ability of vaccine-induced antibodies to inhibit adhesion across these genetically diverse strains suggests that targeting conserved adhesion-associated epitopes may increase immune coverage beyond a single serotype. However, because the in vitro assay measured antibody-mediated adhesion inhibition rather than disease protection, these data support broad anti-adhesion activity rather than definitive broad-spectrum in vivo protection. Heterologous challenge experiments using O1, O78, and additional epidemiologically relevant isolates will therefore be important for determining whether the observed in vitro breadth translates into cross-serotype protection.

The protective efficacy of the FimH-CsgA-OmpA MEFA vaccine was further evaluated in the chicken challenge model. Following APEC-XM (O2) challenge, vaccinated chickens showed improved survival, reduced gross pathological lesions, and decreased recovery of the challenge strain from systemic tissues compared with PBS-treated controls. The fusion-only group showed 100% survival, whereas the MOA-adjuvanted group showed 83% survival. This numerical difference is counterintuitive if the adjuvant is assumed to enhance protection, but the experiment was neither designed nor statistically powered to determine superiority between the two formulations. The difference could reflect sampling variability or formulation-dependent differences in antigen release, distribution, or innate responses. Accordingly, the present data do not support the conclusion that the adjuvant-free formulation is intrinsically superior. Independent replication with a larger sample size and a prespecified direct comparison between formulations is required to determine whether this numerical difference is reproducible.

Whole-cell and attenuated vaccines can elicit broad immune responses but may be limited by safety concerns, variable efficacy, and manufacturing complexity [10,39]. Recombinant subunit vaccines offer defined antigen composition and favorable safety profiles but may provide limited breadth when based on individual antigens [40]. Quantitative comparisons across published APEC vaccine studies should be interpreted cautiously because challenge strains, routes and doses, vaccination schedules, adjuvant formulations, and outcome measures differ substantially. Recombinant Iss vaccination yielded 91.7% survival (11/12) after O78 challenge, compared with 75% (9/12) in non-immunized controls, together with reduced lesion scores and enhanced serum and mucosal antibody responses [19]. An OmpA-containing four-antigen recombinant vaccine (EtsC/OmpA/OmpT/TraT) significantly reduced air-sac and heart–liver lesion scores after APEC O2 challenge; 9/10 vaccinated chickens had no air-sac lesions and 6/10 had no heart–liver lesions, compared with 2/10 and 3/10, respectively, in PBS-treated controls [41]. A recombinant *Salmonella gallinarum* vector expressing APEC type I fimbriae yielded 65% survival (13/20) after O78 challenge and 60% survival (12/20) after O161 challenge, compared with 5% (1/20) and 0% (0/20), respectively, in the corresponding controls [12]. In the present study, the fusion-only and MOA-adjuvanted formulations yielded 100% and 83% survival, respectively, after APEC-XM (O2) challenge, and immune sera inhibited adhesion of five APEC serotypes in vitro. These values should not be interpreted as a head-to-head efficacy comparison. Rather, they provide context for the present construct, whose principal rationale is the combined presentation of conserved adhesion-associated determinants from FimH, CsgA, and OmpA. Heterologous challenge studies are required to determine whether the observed in vitro breadth translates into cross-serotype protection.

Despite these findings, several limitations should be acknowledged. First, although APEC serotypes O1, O2, O8, O78, and O141 were evaluated in the antibody-mediated adhesion inhibition assay, the chicken challenge experiment used only the virulent APEC-XM (O2) strain; therefore, broad in vivo protection remains unproven. Second, the challenge study assessed gross lesions and culture-based recovery of the challenge strain but did not include H&E histopathology, limiting the resolution of the pathological evaluation. Third, the immunization schedule and study design did not establish the minimum protective dose, durability of immunity, persistence of protection, or long-term memory B- and T-cell responses. Fourth, mucosal immunity, particularly secretory IgA (sIgA) at respiratory and intestinal sites, was not directly assessed. Fifth, the construct was designed primarily around B-cell epitopes; T-cell epitope coverage was not deliberately optimized, and the contribution of antigen-specific T-cell subsets remains incompletely characterized. Sixth, additional functional antibody assays, including opsonophagocytic and bactericidal activity, were not performed. Finally, the study was conducted under controlled laboratory conditions and did not address large-scale field efficacy, manufacturing consistency, or commercial feasibility. These limitations define priorities for subsequent validation of the vaccine candidate.

Future studies should evaluate protection against heterologous APEC serotypes, including O1 and O78, and genetically diverse field isolates, with adequately powered replication of the formulation comparison. Challenge studies should incorporate H&E histopathology and quantitative tissue-pathology scoring, together with longitudinal assessment of antibody persistence, memory B- and T-cell responses, and mucosal sIgA. Mechanistic experiments should directly test FimH-dependent innate signaling, including TLR4 involvement, dendritic-cell maturation, and downstream MyD88/TRIF-associated pathways. Further work should define protective T-cell epitopes, evaluate opsonophagocytic and bactericidal antibody functions, optimize antigen dose and delivery route, and assess safety and efficacy under field conditions. These studies will be necessary to determine whether the broad in vitro anti-adhesion activity observed here translates into durable heterologous protection in poultry production settings.

## 5. Conclusions

The FimH-CsgA-OmpA MEFA vaccine combines conserved adhesion-associated determinants from FimH, CsgA, and OmpA in a defined recombinant antigen. Immunization elicited antigen-specific humoral responses, enhanced TNF-α and IL-6 production after antigen restimulation, and serum-mediated inhibition of APEC adhesion across O1, O2, O8, O78, and O141 strains in vitro. In chickens, protection was demonstrated against the tested APEC-XM (O2) challenge, with improved survival, less severe gross lesions, and a lower frequency of challenge-strain recovery than in PBS-treated controls. These findings support further evaluation of the FimH-CsgA-OmpA MEFA as a recombinant APEC vaccine candidate with broad in vitro anti-adhesion activity. However, broad in vivo protection across APEC serotypes has not been established, and the numerical difference between adjuvanted and non-adjuvanted formulations requires independent replication. Heterologous challenge, H&E histopathology, mucosal immunity, long-term immune memory, direct mechanistic analysis of FimH-mediated innate signaling, and field evaluation are important next steps toward defining the translational value of this vaccine candidate.

## Figures and Tables

**Figure 1:**
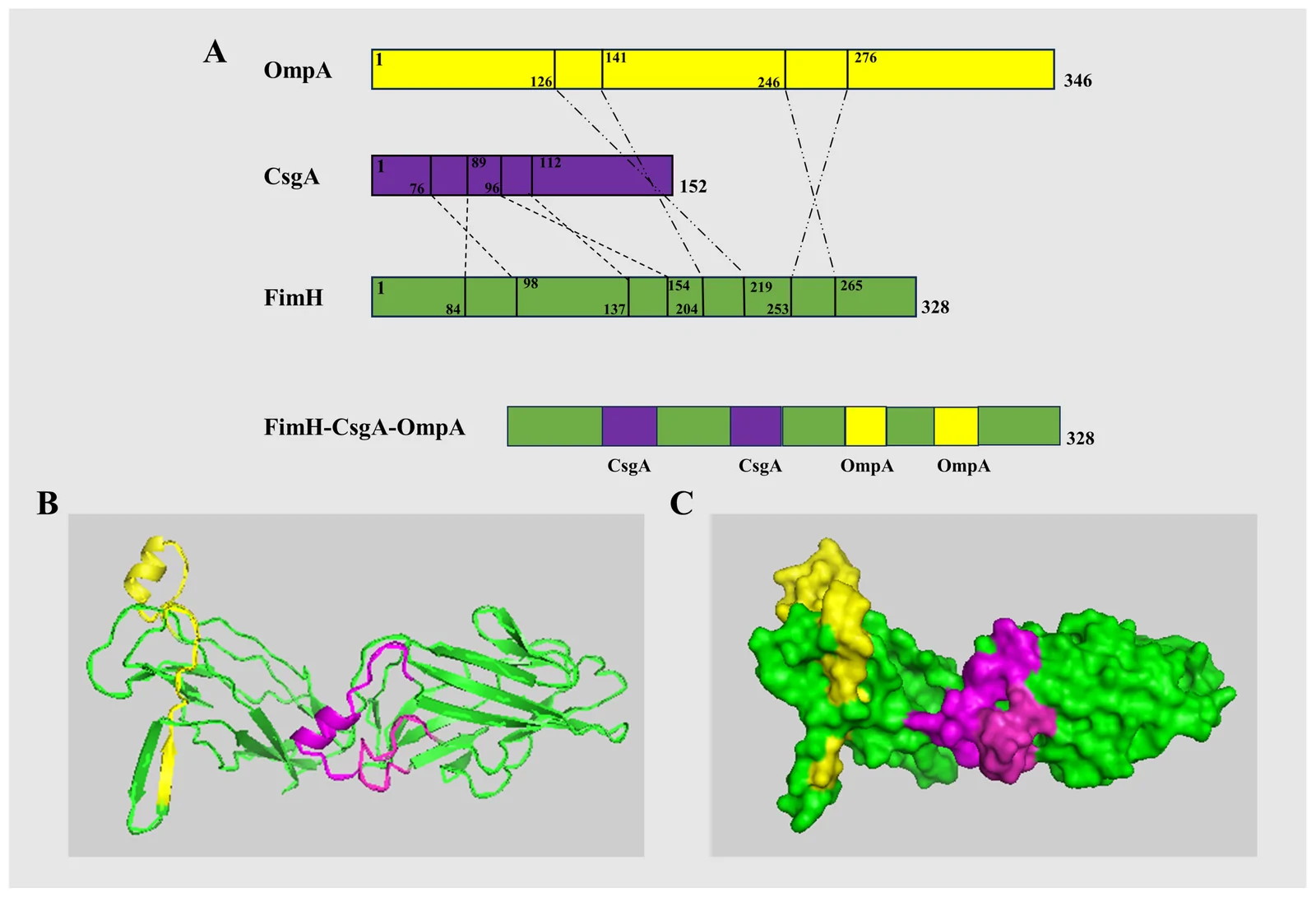
**Design and structural analysis of the FimH-CsgA-OmpA MEFA fusion protein.** (**A**) Schematic representation of the construction of the FimH-CsgA-OmpA MEFA fusion protein. The immunodominant epitopes of CsgA and OmpA were identified and incorporated into the FimH backbone to generate the trivalent fusion protein. The positions of selected epitopes within the parental antigens (OmpA and CsgA) are indicated. Colored boxes represent different antigenic regions, and dashed lines indicate the corresponding positions of the inserted epitopes in the MEFA construct. (**B**) Predicted three-dimensional structure of the FimH-CsgA-OmpA fusion protein. The structural model illustrates the spatial arrangement of the inserted CsgA and OmpA epitopes within the FimH scaffold. (**C**) Surface representation of the predicted FimH-CsgA-OmpA MEFA structure. Different colors indicate the FimH backbone (green), CsgA-derived epitopes (purple), and OmpA-derived epitopes (yellow).

**Figure 2:**
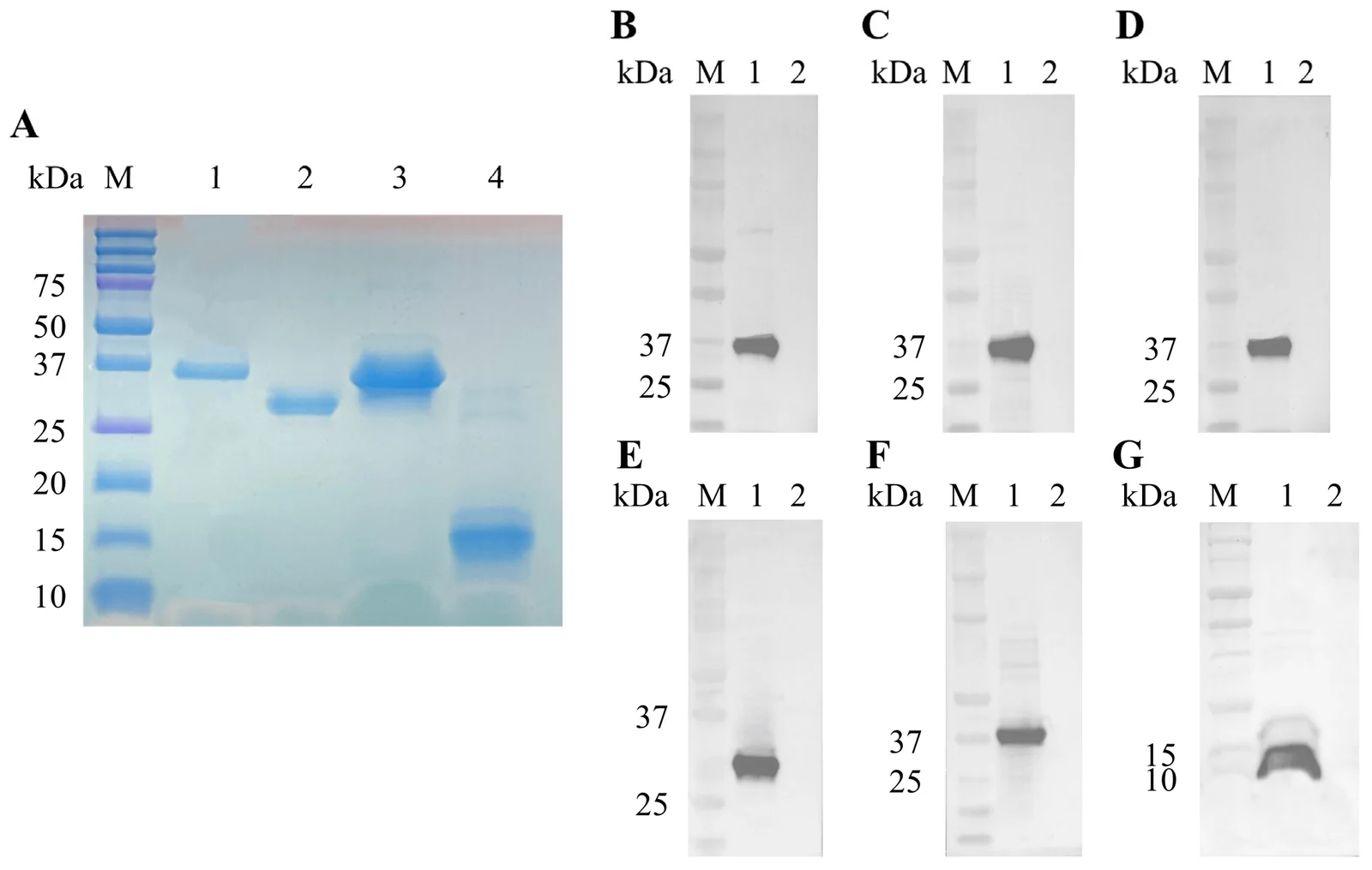
**Expression and identification of the FimH-CsgA-OmpA MEFA fusion protein and recombinant proteins.** (**A**) SDS-PAGE analysis of purified proteins stained with Coomassie brilliant blue. Protein M: Protein Marker 1: FimH-CsgA-OmpA MEFA fusion protein, 2: FimH recombinant protein, 3: CsgA recombinant protein, 4: OmpA recombinant protein. (**B**–**D**) Western blot analysis of the FimH-CsgA-OmpA fusion protein probed with polyclonal antibodies against FimH, CsgA, and OmpA, respectively. M: Protein Marker 1: FimH-CsgA-OmpA MEFA fusion protein, 2: pET-28a (+) vector. (**E**–**G**) Western blot analysis of recombinant FimH, CsgA, and OmpA proteins probed with polyclonal antibodies against the FimH-CsgA-OmpA fusion protein. M: Protein Marker 1: FimH, CsgA, or OmpA recombinant protein, 2: pET-28a(+) vector.

**Figure 3:**
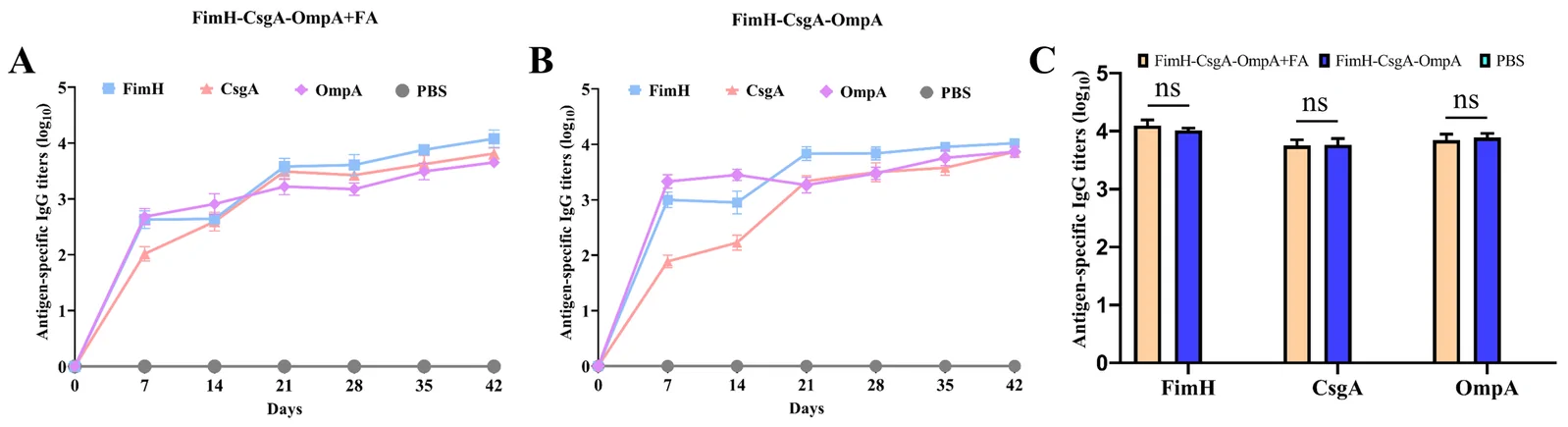
**Antibody responses elicited by immunization with the FimH-CsgA-OmpA MEFA fusion protein in mice.** (**A**,**B**) Kinetics of FimH-, CsgA-, and OmpA-specific IgG antibody responses in mice immunized with FimH-CsgA-OmpA+FA (**A**) or FimH-CsgA-OmpA (**B**). Serum antibody titers were determined by ELISA at the indicated time points. (**C**) Endpoint IgG antibody titers against FimH, CsgA, and OmpA measured after the final immunization. Mice immunized with PBS served as the negative control group. Data are presented as mean ± SD. ns, not significant (*p* > 0.05).

**Figure 4:**
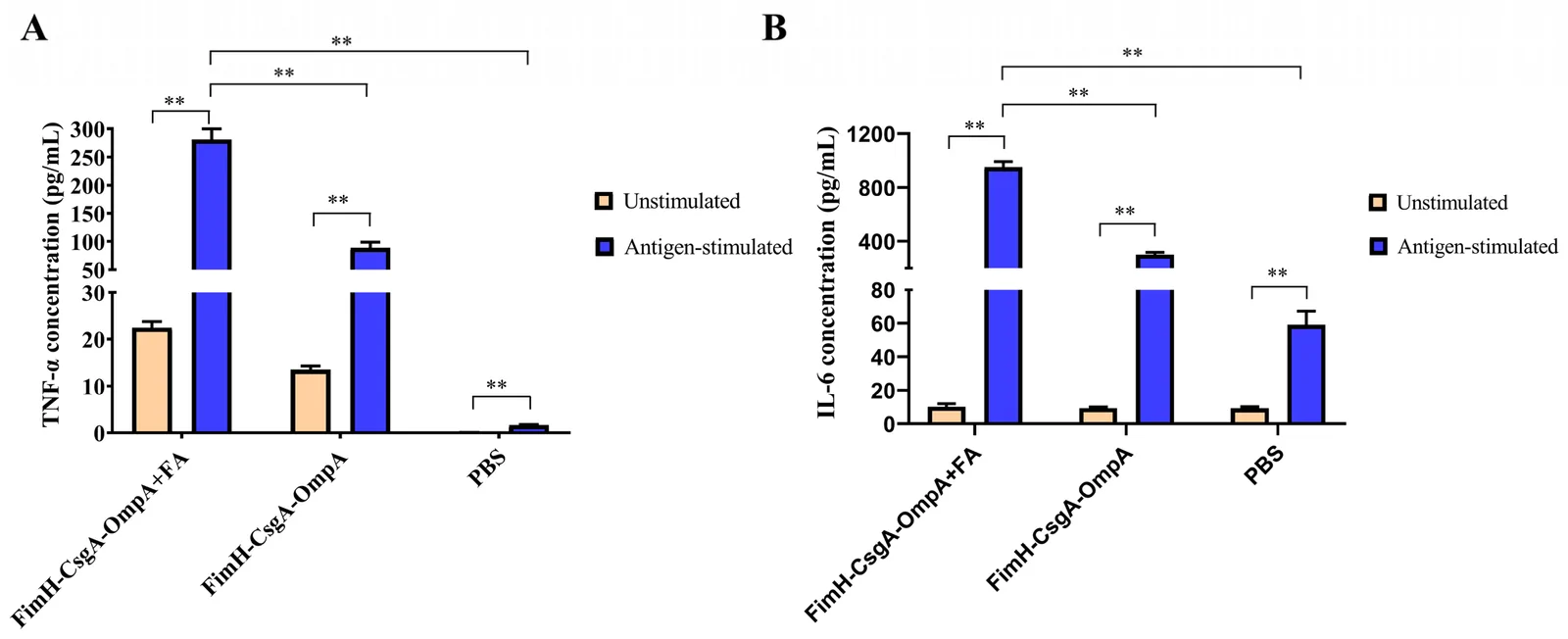
**Antigen-specific TNF-α and IL-6 production induced by immunization with the FimH-CsgA-OmpA MEFA fusion protein.** Splenocytes isolated from immunized mice were re-stimulated in vitro with the recombinant FimH-CsgA-OmpA MEFA fusion protein. The levels of (**A**) TNF-α and (**B**) IL-6 in culture supernatants were quantified by ELISA. Mice were immunized with FimH-CsgA-OmpA+FA, FimH-CsgA-OmpA, or PBS. Data are presented as mean ± SD. ** *p* < 0.01.

**Figure 6:**
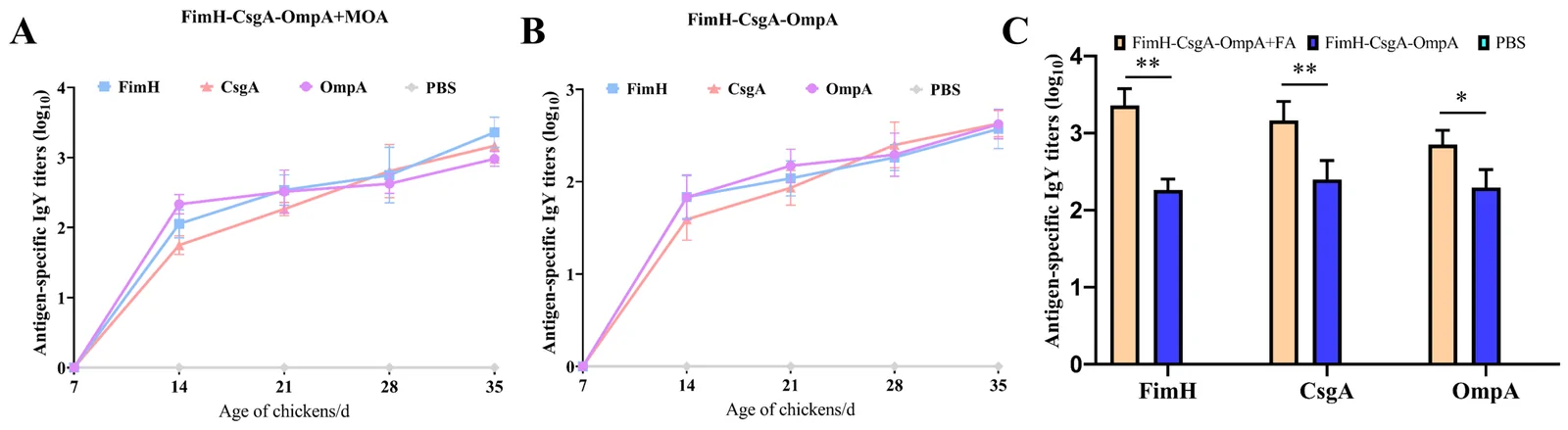
**FimH-, CsgA-, and OmpA-specific IgY antibody responses induced by immunization with the FimH-CsgA-OmpA MEFA fusion protein in chickens.** (**A**,**B**) Kinetics of FimH-, CsgA-, and OmpA-specific IgY antibody responses in chickens immunized with the FimH-CsgA-OmpA MEFA fusion protein formulated with MOA (**A**) or without MOA (**B**). Serum samples were collected at 7, 14, 21, and 28 days post- immunization, and antigen-specific IgY titers were determined by ELISA. PBS-immunized chickens served as the negative control group. (**C**) Endpoint serum IgY titers against FimH, CsgA, and OmpA at 28 days post-immunization in chickens immunized with FimH-CsgA-OmpA+MOA or FimH-CsgA-OmpA. Data are presented as mean ± SD. * *p* < 0.05, ** *p* < 0.01.

**Figure 7:**
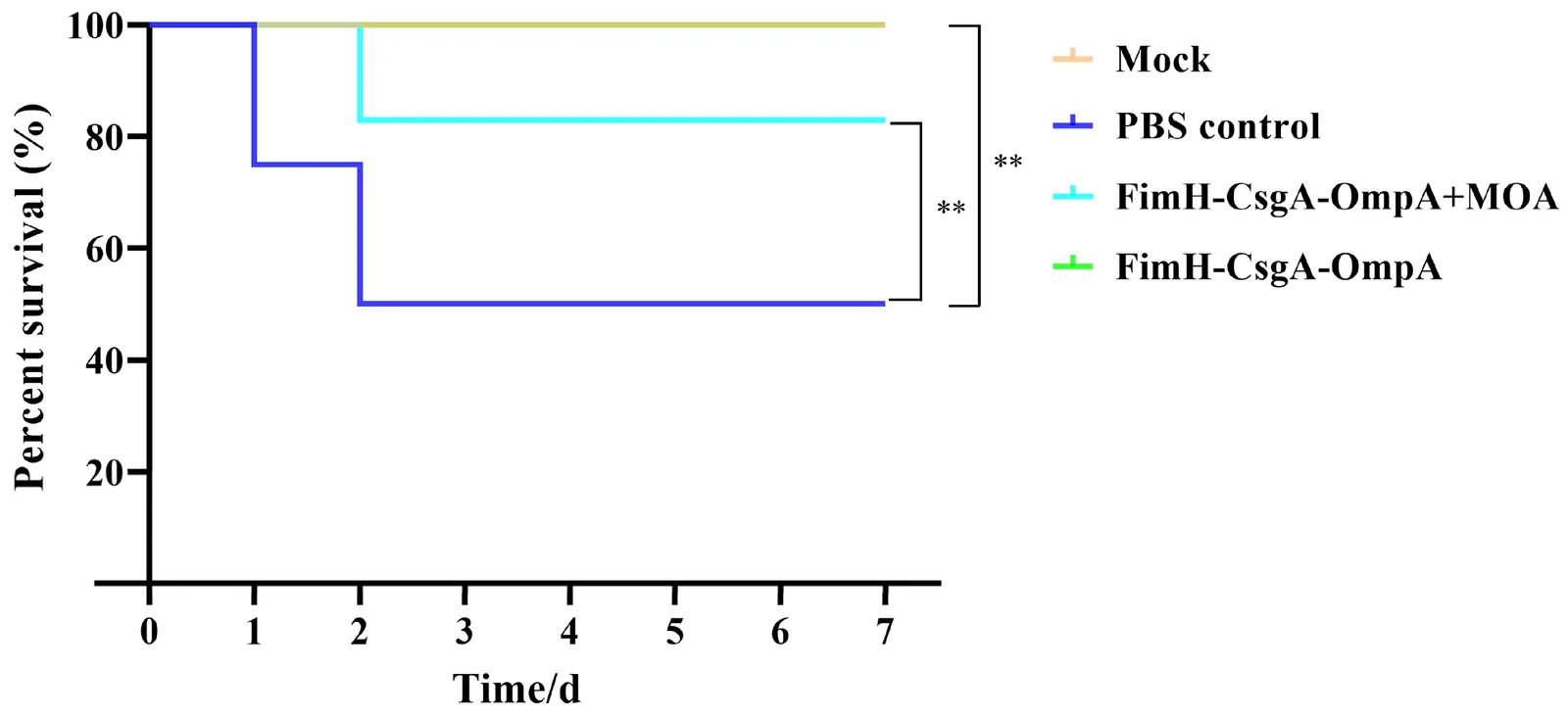
**Immunization improves survival of chickens following APEC challenge.** Kaplan–Meier survival curves of chickens following APEC challenge. Chickens were immunized with FimH-CsgA-OmpA+MOA, FimH-CsgA-OmpA, or PBS and challenged with APEC at 35 days of age. Survival was monitored daily for 7 days post-challenge. The mock group served as an unchallenged control. Survival curves were compared using the log-rank test. ** indicates *p* < 0.01 compared with the PBS control group.

**Figure 8:**
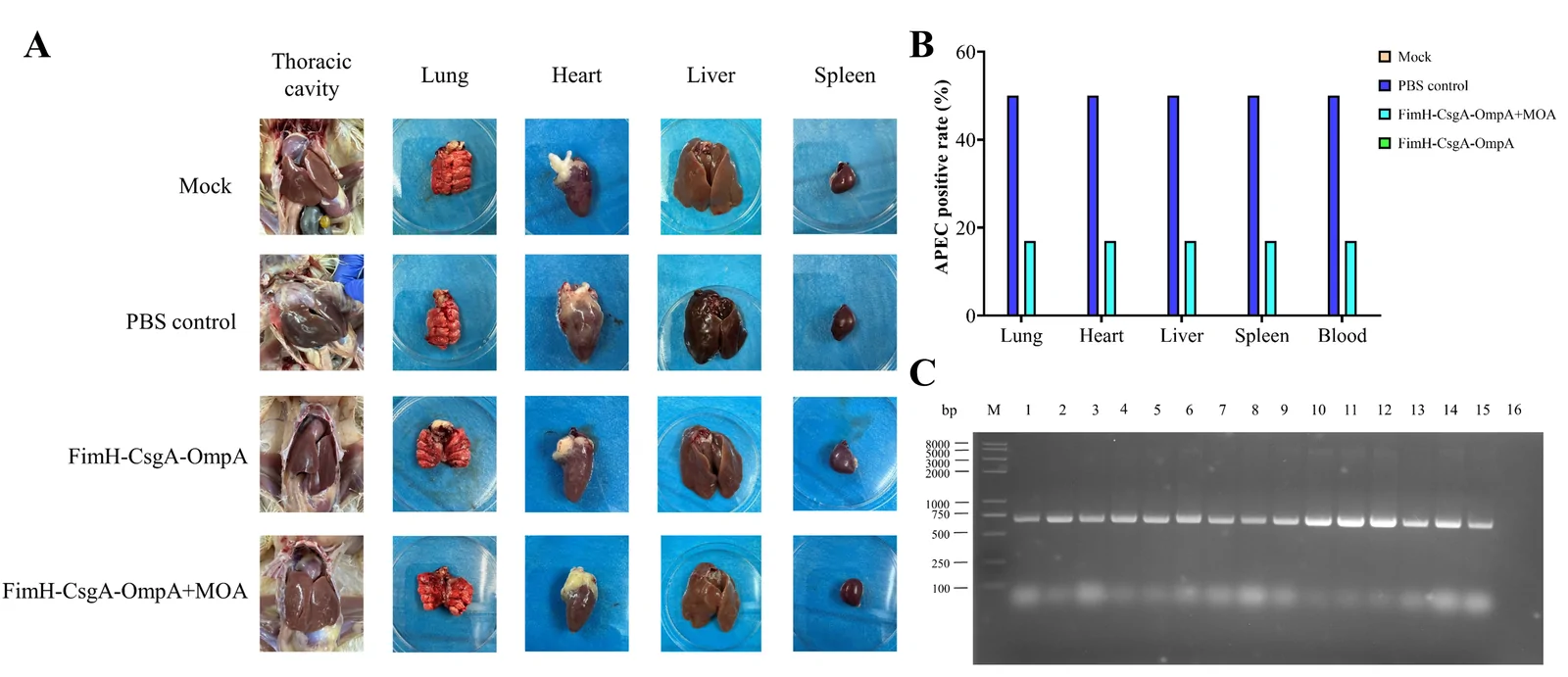
**Immunization alleviates gross pathological lesions and limits APEC-XM recovery in challenged chickens.** (**A**) Gross pathological changes in major organs following APEC-XM challenge. Representative images of organs from the mock, PBS control, FimH-CsgA-OmpA+MOA, and FimH-CsgA-OmpA groups are shown. (**B**) Recovery of APEC-XM from blood and major organs following challenge. Bacterial isolation was performed using blood and organ samples collected from challenged chickens. The proportion of chickens positive for APEC-XM recovery was calculated for each group. (**C**) PCR identification of recovered APEC-XM isolates. Genomic DNA from representative isolates was amplified using primers targeting the *O2* gene fragment. M, DNA marker; lanes 1–15, representative APEC-XM isolates recovered from challenged chickens; lane 16, negative control.

**Table 1:** Primers used in this study.

Primer	Sequences (5′-3′)
*fimH*-F	ATTCGGGATCCATGAAACGAGTTATTACCCTGTTTGC
*fimH*-R	CAGCGTCGACTTATTGATAAACAAAAGTCACGCC
*csgA*-F	ATTCGGGATCCATGAAACTTTTAAAAGTAGCAGCA
*csgA*-R	CAGCGTCGACTTAGTACTGATGAGCGGTCGC
*OmpA*-F	CTAGCTAGCATGAAAAAGACAGCTATCGCGATTG
*OmpA*-R	CAGCGTCGACTTAAGCCTGCGGCTGAGTTAC
*O2*-F	TGGGTGTTCCTGCTTGTTA
*O2*-R	GCGTCCATACTCGCTGTTA

Underlined sequences indicate restriction enzyme sites.

**Table 2:** Distribution and recovery of APEC from blood and major organs of dead and surviving chickens following challenge.

Group	Lung	Heart	Liver	Spleen	Blood
Mock	−	−	−	−	−
Dead chickens	+	+	+	+	+
Survived chickens	−	−	−	−	−

+ indicates positive isolation of APEC-XM from the indicated sample, whereas − indicates no detectable APEC-XM growth by bacterial culture.

## Data Availability

The original contributions presented in this study are included in the article. Further inquiries can be directed to the corresponding authors.
